# Migration of covered stents in thoracic central vein obstruction procedures in patients with hemodialysis: Case report and literature review

**DOI:** 10.3389/fcvm.2022.954443

**Published:** 2022-07-27

**Authors:** Bo Chen, Qiquan Lai, Swalay Fedally, Ziming Wan

**Affiliations:** ^1^Department of Ultrasonography, The First Affiliated Hospital of Chongqing Medical University, Chongqing, China; ^2^Department of Nephrology, The First Affiliated Hospital of Chongqing Medical University, Chongqing, China; ^3^Department of Nephrology, SSRN Hospital, Mauritius, Mauritius

**Keywords:** stent displacement, stent migration, hemodialysis, literature review, thoracic central vein obstruction

## Abstract

**Objective:**

The objective of the study is to present a case of hemodialysis in which the covered stent that had migrated into the right ventricle was retrieved by exploratory thoracotomy, and to review the literature on the diagnosis and treatment of stent migration in thoracic central vein obstruction (TCVO) procedures for hemodialysis patients.

**Method:**

A systematic search of the PubMed database was performed to identify clinical presentations, imaging strategies, stent types, and treatment modalities for stent migration in hemodialysis patients.

**Results:**

A total of 14 case reports on stent migration in TCVO procedures for hemodialysis patients were included and analyzed. Ten cases included migration to the cardiac chambers and the remainder migration to the pulmonary artery. The common symptoms of stent migration in TCVO procedures are reported to be chest pain and dyspnea, while three of the cases studied involved no symptoms. Echocardiography, chest X-ray, and computed tomography are the commonly used methods for the diagnosis of stent migration and identification of the precise positioning of the stent. Stent migration to the right subclavian or innominate veins was the most prevalent case (seven cases). All were bare stents. Seven cases involved retrieval by interventional surgery, while four cases involved retrieval by open heart surgery. However, there were three cases in which the “wait-and-see” approach was adopted since the patients were asymptomatic.

**Conclusions:**

Stent migration in TCVO procedures is a rare but extremely serious complication. The causes are not fully understood. The current treatment strategies include interventional surgery, open heart surgery, and the “wait-and-see” approach.

## Introduction

Thoracic central vein obstruction (TCVO) is a common complication that occurs with prolongation of the survival time of hemodialysis patients, and it seriously affects the function of vascular access or the quality of hemodialysis (1–3). Percutaneous transluminal angioplasty (PTA) is the first treatment option for TCVO, while other possible treatment options include percutaneous transluminal vascular stent placement. Intravascular stents provide a useful and safe means for preventing recurrent stenosis or occlusion and ensuring patency in TCVO (4–6). However, stent utilization entails a number of inherent complications including stent fracture, stent migration, infection, and in-stent stenosis (7).

Stent migration is a rare complication, with the incidence reported to be as low as 2%−3% (8–10). However, stent migration in TCVO procedures could result in serious consequences including cardiac perforation, pericardial tamponade, pulmonary embolism, tricuspid valve regurgitation, and heart failure (11). To the best of our knowledge, only a few studies have reviewed the characteristics of the cases of stent migration in TCVO procedures, especially those pertaining to cases of covered stent migration. Therefore, this study presents a new case of the procedure on a 65-year-old man in whom the covered stent located at the confluence of the right subclavian and innominate veins migrated to the right ventricle following stent placement and resulted in acute tamponade, and a review of the published literature on previously reported cases.

### Case report

**Figure 1:**
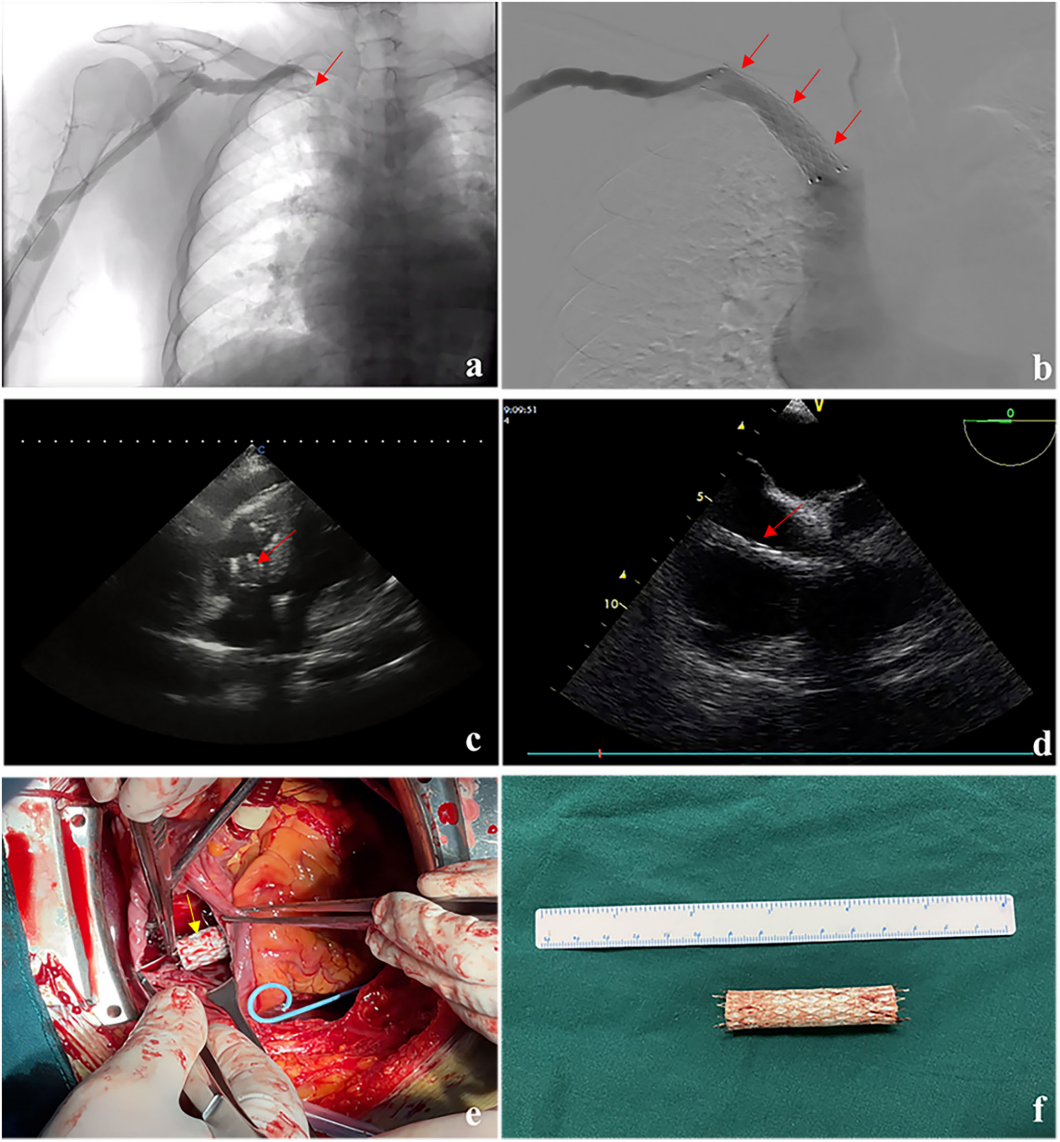
Imaging data and intraoperative data of the patients: **(a)** stenosis (red arrow) of the confluence of the right subclavian vein and the innominate vein in a digital subtraction angiography examination, **(b)** stent (red arrow) placement in central vein stenosis, **(c)** transthoracic echocardiography showing pericardial effusion and a stent (red arrow) in the right ventricle, **(d)** transesophageal echocardiography indicating a segment of stent migration (red arrow) in the right ventricle, **(e)** the tail of the stent (yellow arrow) was found to have been inserted into the atrial wall during the operation, and **(f)** a retrieved covered stent.

A 65-year-old male had been undergoing hemodialysis with an arteriovenous fistula placed on his right wrist for 11 years. He was transferred to our hospital with a main complaint of swelling of the right hand, and he had experienced high venous pressure with no clear cause around 4 months earlier. The patient had a history of right internal jugular vein catheterization. The digital subtraction angiography (DSA) examination revealed stenosis at the confluence of the right subclavian vein and the innominate vein (Figure 1a). A 13.5 × 60-mm covered stent (Fluency Plus, Bard, United States) had to be used for the stenosis following PTA because of significant elastic recoiling of the lesion. Following this, no residual stenosis or intimal tear was observed, and the stenosis was relieved (Figure 1b). However, 1 h later, the patient experienced chest pain without a clear cause accompanied by pallor, sweating, irritability, nausea, and vomiting.

On physical examination, the heart rate recorded was 115 times/min, respiratory rate was 26 times/min, and blood pressure was 81/55 mmHg. Cyanosis of the lip and low heart sounds were detected. Following an echocardiography examination, pericardial effusion and a stent in the right atrium and ventricle were identified (Figure 1c). It was deemed that the stent had fallen to the right side of the heart, which led to pericardium tamponade.

Pericardiocentesis was then immediately conducted under the guidance of ultrasound, with 270 ml of pericardial effusion extracted, and the patient's symptoms were relieved. The thoracotomy and transesophageal echocardiography procedures indicated that the stent had migrated to the right ventricle (Figure 1d) with its tail located at the convergence of the inferior vena cava (IVC) and the right atrium. No mural thrombus was found in the right atrium or the right ventricle, while only a small number of hematomata were found at the convergence of the IVC and the right atrium after the heart was exposed. The right atrium was opened, and it was clear that the tail of the covered stent was inserted into the wall of the right atrium (Figure 1e) and was close to the convergence of the IVC. The other side of the covered stent floated in the right ventricle across the tricuspid valve. The covered stent was successfully retrieved (Figure 1f), and the crevasse at the convergence of the IVC was repaired. The patient recovered successfully and resumed normal hemodialysis.

## Literature review

### Methods

A PubMed search of literature published from 1975 to February 2022 was conducted along with a bibliographic search of published articles to identify clearly documented cases of stent migration in TCVO procedures for hemodialysis patients. All available data were reviewed including in terms of clinical presentation, imaging strategy, stenttype, and treatment.

## Results

A total of 14 case reports on stent migration in TCVO procedures for hemodialysis patients were included and analyzed. Ten of the cases (12–21) involved migration to the cardiac chambers (Table 1), while the remainder (22–25) involved migration to the pulmonary artery (Table 2). The common symptoms of stent migration in TCVO procedures were reported to be chest pain and dyspnea. However, three of the studied cases (19, 23, 25) involved no symptoms.

**Table 1:** Clinical and technical aspects of 11 patients with misplaced or dislodged stents in the cardiac chambers.

**Author**	**Year**	**Age**	**sex**	**Onset timing (hour, day, month)**	**Clinical presentation**	**Imaging strategies**	**Location of lesion**	**Size of stent**	**Stent type**	**Retrieval technique**	**Position of stent migration**
Kaneko et al. (12)	2008	78	M	2 days later	General fatigue and bradycardia	Echocardiogram	Left innominate vein	10 mm × 39 mm	Wallstent	Snaring stent directory and implanted it in the iliac vein	Right ventricle
Poludasu et al. (13)	2008	74	M	1 year later	Chest pain	Echocardiogram	Superior vena cava	14 mm × 60 mm	Nitinol symphony	Open heart surgery	Right ventricle
Bagul et al. (14)	2008	78	F	14 days later	Not description	Chest X-ray	Superior vena cava	10 mm × 46 mm	Wallstent	Snaring stent directory and surgically removal from the groin	Right atrium
Kang et al. (15)	2011	40	M	Immediately	Dyspnea on exertion	Echocardiogram	Right subclavian vein	14 mm × 60 mm	Zilver	Open heart surgery	Right ventricle
Bani-Hani et al. (16)	2012	88	F	4 h later	Retrosternal chest pain	Echocardiogram	Junction of the right subclavian and innominate veins	12 mm × 40 mm	Protégé	Retrieval via the right common femoral vein with a snare	Right ventricle
Daprati et al. (17)	2012	72	Not know	Not description	Not description	Echocardiogram	Right subclavian vein	Possibly 12 mm × 60 mm	Wallstent	Snaring stent directory and surgically removal from the groin	Right atrium
Cohen et al. (18)	2012	88	F	4 months later	Not description	Chest X-ray and echocardiogram	Left innominate vein	15 mm × 40 mm	Wallstent	Open heart surgery	Right ventricle
Khaddash et al. (19)	2016	56	F	Not description	No symptom	Echocardiogram	Right subclavian vein	Not know	Not know	Wait-and-see	Right atrium
Vijayvergiya et al. (20)	2020	42	F	Not description	Not description	Echocardiogram	Superior vena cava	16 mm × 60 mm	Wallstent	Another stent was deployed from the SVC to the inferior vena cava	Right atrium
Khazi et al. (21)	2021	37	F	4 h later	Shortness of breath with central chest pain	Echocardiogram and computed tomographic	Superior vena cava	Not know	Not know	Open heart surgery	Right atrium

**Table 2:** Clinical and technical aspects of four patients with misplaced or dislodged stents in the pulmonary artery.

**Author**	**Year**	**Age**	**sex**	**Onset timing (hour, day, month)**	**Clinical presentation**	**Imaging strategies**	**Location of Lesion**	**Size of stent**	**Stent type**	**Retrieval technique**	**Position of stent migration**
Gray et al. (22)	1994	79	F	Immediately	No description	Chest X-ray	Right subclavian and innominate veins	30 mm	Palmaz	Balloon-assiated reposition, snaring stent and deployment to the iliac vein	Left pulmonary artery
Sharma et al. (23)	2002	70	M	4 months later	No symptom	Chest X-ray	Right subclavian vein	12 mm	Wallstent	Wait-and-see	Right pulmonary artery
Sugahara et al. (24)	2012	36	M	Immediately	No description	Digital subtraction angiography	Right innominate vein	7 mm × 40 mm	SMART	Balloon-assiated reposition, snaring stent and deployment to the femoral vein, and surgically removal from the groin	Left pulmonary artery
Kakisis et al. (25)	2014	83	M	1 day later	No symptom	Chest X-ray and computed tomography	Left innominate vein	10 mm × 38 mm	Omnilink	Wait-and-see	Left lower lobe Pulmonary artery

In terms of imaging strategies, echocardiogram, chest X-ray, and computed tomography examinations were the commonly used methods for the diagnosis of stent migration and identification of the precise positioning of the stent. There were seven cases of stent migrating to the right subclavian or innominate veins, four cases of migration to the superior vena cava (SCV), and three cases of migration to the left innominate vein. All were bare stents, and no covered stent migration was reported.

In terms of treatment strategy, seven cases involved retrieval by interventional surgery with the help of a snare, and the stent ultimately had to be surgically retrieved from the groin by opening the femoral vein or expanding the stent with the help of a balloon to immobilize it in the wall of the femoral vein. Four cases involved retrieval by open heart surgery, including two cases in which retrieval by interventional surgery had failed (15, 18). However, in three cases, the “wait-and-see” approach was adopted since the patients were asymptomatic.

## Discussion

Nowadays, the use of stent placement to enhance the patency rate of vascular access in hemodialysis patients has been increasing, especially in terms of TCVO (26). As such, the occurrence rate of stent migration is likely to increase (8–10). A total of 14 reports on stent migration in TCVO procedures were identified and reviewed. All the reviewed cases involved the use of bare stents. This study presents the first report on covered stent migration in TCVO procedures for hemodialysis patients.

### Comparison with previous studies

To the best of our knowledge, this is the first study to review the literature related to stent migration in TCVO procedures for hemodialysis patients. Only one previous study involved a review of 29 case reports and two short case series of venous stent migration; it detailed 54 events of venous stent migration (27). Here, the authors reported the treatment strategies of all cases and preliminarily analyzed the cause of stent migration. However, the causes and differences among the cases of central venous stent migration in hemodialysis patients have not yet been discussed independently.

Hemodialysis patients have certain particularities in terms of different hemodynamics for the establishment of vascular access. In addition, previous researchers have not discussed the advantages and disadvantages of various treatments in-depth or have subsequently provided recommendations.

### Causes of stent migration

Stent migration can be caused by multiple factors including positional distinctiveness of the venous stent, hemodynamics, properties of the stent, and improper selection of stent (7). In addition, the diameter of the central vein gradually increases as it gets closer to the heart, and it also changes with respiratory movement (10). In terms of self-expanding stent deployment, if prior pre-dilation of a stenotic segment is a risk factor remains debatable (28, 29). These causes were confirmed by a review of the literature.

First, most of the stents in the reported cases were located in the right subclavian vein or the right innominate vein (15–17, 19, 22–24). In our opinion, this was a result of the particularity of the anatomical position of the confluence of the right subclavian vein and the right innominate vein, with the larger angle at almost 90°, meaning it was more likely to migrate compared with other positions.

Second, it can be difficult to select the correctly sized stent, since the expansion of the vein occurs far more easily than that of the artery. This is particularly true in the case of hemodialysis patients, with the central vein more likely to expand and distort in the process of arterialization of the central vein due to the vascular access, while its shape is subjected to respiratory motion and external compression from surrounding structures since this vein is more pliable (30). This phenomenon leads to some difficulties in terms of the accuracy of measurement, with venography examinations potentially mischaracterizing venous stenosis. As such, interventionalists have recommended the deployment of an oversized stent graft with a diameter of between 18 and 22 mm (31). However, no case in which such an oversized stent was adopted is reported in the existing literature, likely because an oversized stent may more easily stimulate intimal hyperplasia.

Third, the length of the stent should cover the lesion with at least 10 mm free at both the proximal and the distal directions to the lesion, with 60% of the stent ideally located at the distal end of the lesion and 40% at the proximal end to avoid the migration of the stent toward the latter end due to extrusion (31, 32). However, it can prove difficult to implant the stent for TCVO in hemodialysis patients using the above principle, especially for lesions of the subclavian or innominate vein„ since covering up important branch veins such as the cephalic vein arch and jugular vein must often be avoided. In addition, the above-mentioned reasons may be causes of stent migration within a few hours both in our case study and in previously reported cases.

### Treatment strategies

The treatment strategies for the stent migration in TCVO procedures include retrieval by interventional surgery, surgical removal, and “leave it alone” and “wait-and-see” approaches. Interventional surgery includes extracting the stent to the femoral vein using a “gooseneck” device, opening the femoral vein, and retrieving the stent or the implanted stent in the vein with optimally sized balloon inflation. However, in the case of stent migration to the pulmonary artery, the better option may be to withdraw the stent by balloon-assisted reposition. There was also one case in which the stent was immobilized with another stent from the SVC to the IVC through the migrated right atrial stent using the bridging stent technique (20).

The literature review revealed that the success rate of interventional surgery for retrieving a migrated stent is high, and that this is the first option for most cases. However, in two cases, the attempt to retrieve the migrated stent using a gooseneck device failed, but the retrieval was ultimately achieved by surgical thoracotomy (15, 18). In terms of open heart surgery, this can present an important and effective means in certain situations. In our case study, the thoracotomy method was adopted to retrieve the migrated stent based on the characteristics of the fluency stent. Specifically, it was deemed that the interventional procedure carries a high risk of vessel wall or heart perforation since the tip of the stent was extremely sharp, which was also confirmed during the operation. The “wait-and-see” approach was also adopted for a small number of asymptomatic patients (19, 23, 25). However, the long-term prognosis of the cases was not clear.

### Limitations

The current study involved several limitations. First, the risk of publication bias was strong given the paucity of reported data. In addition, since many migration events are potentially asymptomatic, the possibility that stent migration may not have been reported was high, meaning the sample size was comparatively small. Second, while by literature review and through our own clinical experience the causes of stent migration were discussed, it proved difficult to identify an appropriate method with which to verify them. Third, while various treatment options were discussed and analyzed, there exists no definitive evidence of their safety and effectiveness.

### Future research directions

More accurate assessment of the thoracic central vein and further guidance for selection of an appropriately sized stent are key points for future research. The recent years have witnessed an increase in the use of intravascular ultrasound in venous applications as endovascular therapy for central venous diseases since it is more sensitive in terms of detecting and characterizing stenotic diseases and assessing the intraluminal parameters of the central vein (30). A number of studies have reported its application, with remarkable effect, in the treatment of central venous disease in dialysis patients (33–35). However, further prospective randomized controlled trials are required for verification. Additionally, while PTA and stent placement are, at present, the most favorable options for TCVO procedures in hemodialysis patients, both involve certain limitations including obstruction of important branches and stent migration. As such, better treatment options for TCVO must be explored in the future. A number of studies have reported that first rib resection has excellent assisted primary and secondary patency rates when combined with endovascular techniques for the treatment of TCVO at the thoracic outlet in hemodialysis patients (36, 37).

## Conclusion

In TCVO procedures, stent migration is a rare but extremely serious complication. The causes are not yet fully understood, and the current treatment strategies include interventional surgery, open heart surgery, and the “wait-and-see” approach.

## Data availability statement

The original contributions presented in the study are included in the article/supplementary material, further inquiries can be directed to the corresponding author.

## Author contributions

SF was responsible for polishing the language of manuscript. All authors contributed to the article and approved the submitted version.

## Funding

This work was supported by Science-Health Joint Medical Research Project of Chongqing (Number: 2020FYYX064).

## Conflict of interest

The authors declare that the research was conducted in the absence of any commercial or financial relationships that could be construed as a potential conflict of interest.

## Publisher's note

All claims expressed in this article are solely those of the authors and do not necessarily represent those of their affiliated organizations, or those of the publisher, the editors and the reviewers. Any product that may be evaluated in this article, or claim that may be made by its manufacturer, is not guaranteed or endorsed by the publisher.
